# Supplementary material for: Space–Time Clustering Characteristics of Malaria in Bhutan at the End Stages of Elimination
Source: Int J Environ Res Public Health. 2021 May 22;18(11):5553. doi: 10.3390/ijerph18115553 (PMC8196969; doi:10.3390/ijerph18115553)
Supplement: Supplementary file 1 [file ijerph-18-05553-s001.zip › ijerph-1202955-supplementary.pdf]

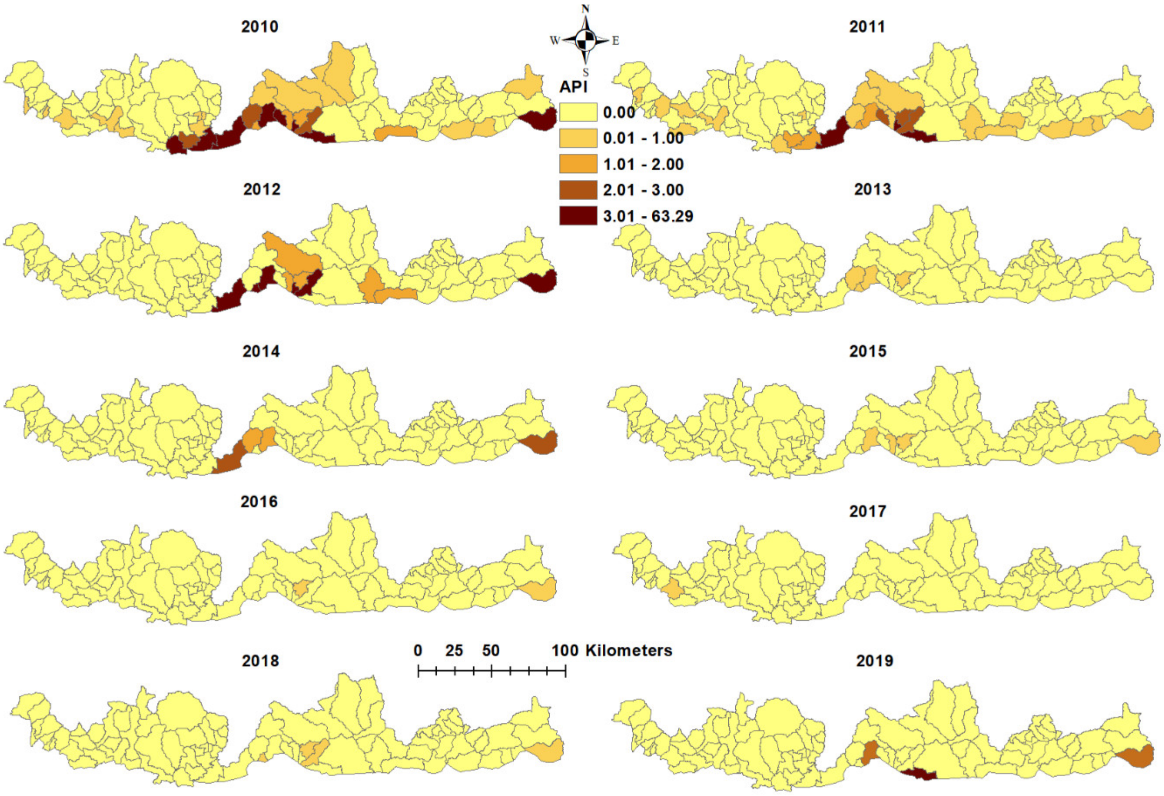

Figure S1. Annual parasite incidence of *Plasmodium falciparum*.

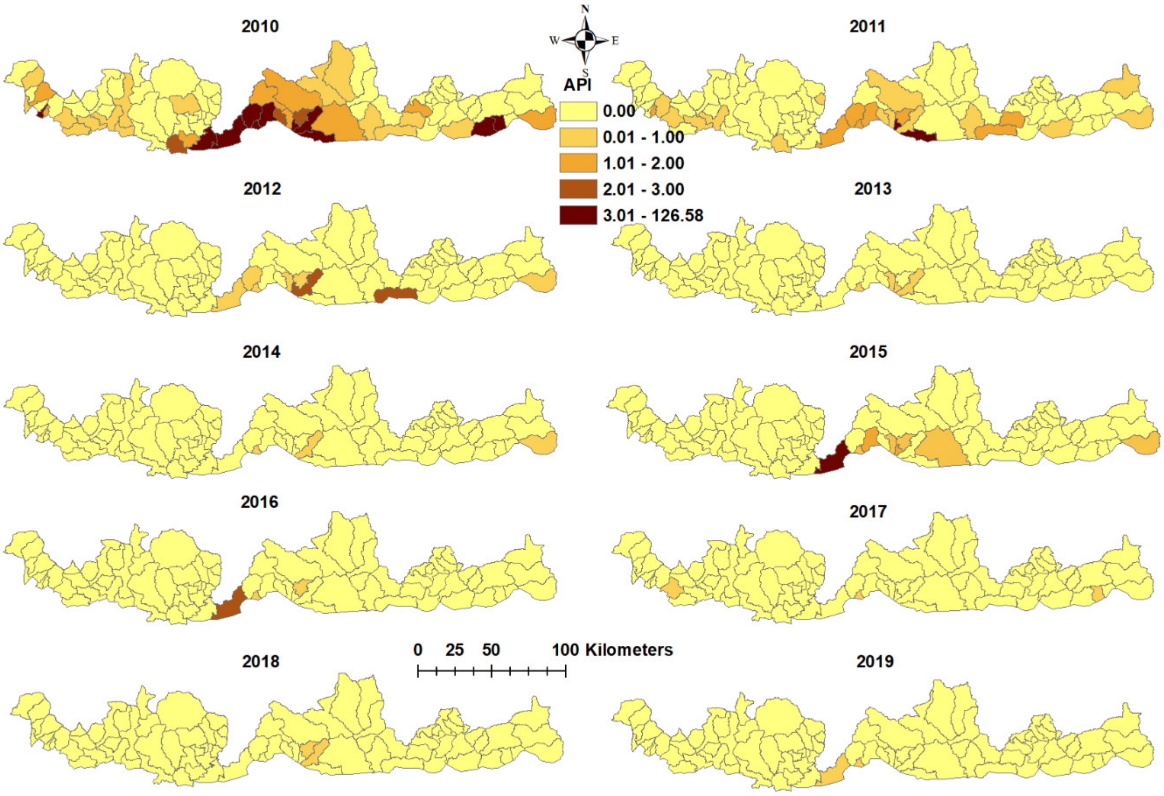

Figure S2. Annual parasite incidence of *Plasmodium vivax*.
